# 3-Dimensional morphological characterization of neuroretinal microglia in Alzheimer’s disease via machine learning

**DOI:** 10.1186/s40478-024-01898-6

**Published:** 2024-12-24

**Authors:** Wissam B. Nassrallah, Hao Ran Li, Lyden Irani, Printha Wijesinghe, Peter William Hogg, Lucy Hui, Jean Oh, Ian R. Mackenzie, Veronica Hirsch-Reinshagen, Ging-Yuek Robin Hsiung, Wellington Pham, Sieun Lee, Joanne A. Matsubara

**Affiliations:** 1 Faculty of Medicine, The University of British Columbia, Vancouver, BC Canada; 2 Department of Ophthalmology and Visual Sciences, The University of British Columbia, 2550 Willow St. Room 375, Vancouver, BC V5Z 3N9 Canada; 3 Department of Cellular and Physiological Sciences, The University of British Columbia, Vancouver, BC Canada; 4 Department of Pathology and Laboratory Medicine, The University of British Columbia, Vancouver, BC Canada; 5 Vanderbilt University School of Medicine, Vanderbilt University Institute of Imaging Science, Nashville, TN USA; 6 Simon Fraser University School of Engineering Science, Burnaby, BC Canada; 7 Mental Health and Clinical Neurosciences, School of Medicine, University of Nottingham, Nottingham, UK; 8 Division of Neurology, Department of Medicine, The University of British Columbia, Vancouver, BC Canada

**Keywords:** Alzheimer’s disease, Neuroretinal microglia, 3-Dimensional morphology, Microglia morphology, Microglia count, Microglia size, Machine learning, Phagocytic cups

## Abstract

Alzheimer’s Disease (AD) is a debilitating neurodegenerative disease that affects 47.5 million people worldwide. AD is characterised by the formation of plaques containing extracellular amyloid-β (Aβ) and neurofibrillary tangles composed of hyper-phosphorylated tau proteins (pTau). Aβ gradually accumulates in the brain up to 20 years before the clinical onset of dementia, making it a compelling candidate for early detection of AD. It has been shown that there is increased deposition of Aβs in AD patients’ retinas. However, little is known about microglia’s ability to function and clear Aβ within the retina of AD and control eyes. We labelled microglia with ionised calcium-binding adaptor molecule 1 (IBA-1) in AD and age-matched control donor retinas. We then used interactive machine learning to segment individual microglia in 3D. In the temporal mid-peripheral region, we found that the number of microglia was significantly lower in AD retinas compared to controls. Unexpectedly, the size of the microglia was significantly larger in the AD retinas compared to controls. We also labelled retinal microglia for Cluster of Differentiation 68 (CD68), a transmembrane glycoprotein expressed by cells in the monocyte lineage and a marker of phagocytic activity and activated microglia. The size of CD68 + cells was statistically different between AD and control microglial, with CD68 + cells being larger in AD. In contrast, there was no difference in either size or shape for CD68- microglia between the two groups, suggesting an important difference in the active states of CD68 + microglia in AD retina. There was also significantly increased CD68 immunoreactivity in individual microglia within the AD group. Overall, this study reveals unique differences in the size and activity of the retinal microglia, which may relate to their potential chronic activation due to increased levels of Aβs in the AD retina.

## Introduction

Alzheimer’s Disease (AD) is a neurodegenerative disease that affects older adults, especially those over the age of 65 [1]. The risk of developing AD rises significantly with age [1, 2]. Dementia affects 55 million people worldwide, with AD contributing to 60–70% of all cases [3]. The prevalence is 5–7% in most countries, and the global annual cost incurred by dementia is $1.3 trillion USD [3, 4]. Despite AD first being described more than 120 years ago, there has been no effective treatment that can cure or slow the cognitive decline or severity of dementia seen in AD [2]. However, recent FDA-approved drugs (Aducanumab, Donanemab, and Lecanemab) have been shown to remove amyloid-β (Aβ) from the brain and reduce cognitive and functional decline in people living with early AD [5, 6]. The prevalence and socio-economic impact of AD accentuates the need for further research.

AD is characterised by the formation of plaques containing extracellular Aβ and neurofibrillary tangles composed of hyper-phosphorylated tau proteins (pTau) in the central nervous system (CNS). Given that Aβ gradually accumulates in the brain starting as early as 20 years before the clinical presentation of dementia, it is a compelling candidate for early detection of AD. Unfortunately, conventional structural imaging such as Computed Tomography (CT) and Magnetic Resonance Imaging (MRI), though not invasive, lack sensitivity [7, 8]. Alternatively, Positron Emission Tomography (PET) with ^11^C-radioisotope 11 labelled Pittsburgh compound B—which detects cerebral Aβ deposition—has been shown to distinguish Aβ levels in AD and non-AD brains, but this method is costly, invasive, and not feasible to deploy in community settings [9].

The retina shares many features with the brain and exhibits manifestations of AD, such as Aβ deposition, highlighting its potential as a tool for assessing AD development and progression. Being an extension of the CNS, the retina can be imaged and studied using non-invasive methods such as Optical Coherence Tomography (OCT) and Scanning Laser Ophthalmoscopy (SLO) [10, 11].

One of the essential cells that helps maintain homeostasis within the retina and the CNS is microglia. Microglia serve a critical function in the retina and CNS, acting as the primary immune defence cell, in constant surveillance for signs of distress. Their morphology reflects their state of activation; when not activated, they assume a ramified morphology, whereas they become de-ramified and amoeboid when activated—triggered by detection of any foreign particles or damage-associated molecular patterns (DAMPs). When activated, they phagocytose cellular debris, pathogens, and dead neurons, and thereby maintain a healthy neural environment. Microglia are also activated by and phagocytose Aβ peptides that are toxic to surrounding neurons [12–14]. However, prolonged Aβ exposure can lead microglia to dysregulation and death, which leads to compromised Aβ clearance, neurotoxicity, and neurodegeneration [15, 16].

Though microglia have distinct morphology for their active and resting states, there exists a spectrum of morphologies between its states. Guo et al. [17] postulated that microglia change shape from least active to most active in the following stages: ramified, hyper-ramified, activated, and then amoeboid. There exists manual and machine learning based approaches to classifying microglia morphology. Though manual image analysis has been the standard for many years in biology, it is prone to observer subjectivity whereas machine learning approaches offer a more objective assessment with appropriate overhead if used properly. The current machine learning approaches use cluster analysis, or more recently support vector machines which were trained on large datasets to classify microglia. A recent review by Reddaway et al. [18] provides an overview of microglial morphometric analysis approaches in the literature.

In this study, we examine IBA-1 and CD68-labelled microglia morphology in age-matched control and AD human retinas. Rather than focusing on classifying microglia, we accurately delineated 3D microglia using voxel segmentation with active learning in Ilastik [19], coupled with robust post-processing and quality control steps, and computed various shape parameters for group comparison. Active learning [20] is machine learning approach where the algorithm can be supervised interactively. This method offers several advantages: compared to manual labelling, an automated method can provide detailed segmentation of 3D microglia with reduced rater bias and time cost. Given the small sample size of donor retinal tissues with pathology and significant inter-subject variability even with consistent sample processing, immunofluorescence labelling, and image acquisition parameters, using an off-the-shelf segmentation tool was impractical. Additionally, generating training data through detailed manual segmentation of 3D microglia is both difficult and time-consuming. Ilastik’s active learning workflow for voxel segmentation enabled optimized data utilization through supervised training and iterative performance improvement based on real-time results. The resulting semantic segmentation was fed into a custom Python pipeline for post-processing and identification of individual microglia.

To date, no study has thoroughly examined 3D microglial morphology and activity state in the context of AD in the retina. Given that the AD retina displays elevated Aβ deposition, and activated microglial cells are involved in Aβ clearance, dysfunctional Aβ clearance in the AD retina may be due to an impaired activation and a reduced population of microglial cells.

## Methods

### Neuropathological assessment of Alzheimer’s disease

This study was approved by the clinical ethics research board of the University of British Columbia and strictly adhered to the Declaration of Helsinki. AD retinas were dissected from the post-mortem eyes of donors whose brains were neuropathologically assessed in accordance with the National Institute on Aging Alzheimer’s Association guidelines for the neuropathologic assessment of Alzheimer’s disease [21]. Sample demographics and diagnostic details can be found in Table 1. The Department of Pathology and Laboratory Medicine at Vancouver General Hospital (VGH) provided the eyes from AD donors. Control eyes were provided by the Eye Bank of British Columbia. Exclusion criteria for the control eyes were CNS disorders including AD, Parkinson’s disease, multiple sclerosis, and amyotrophic lateral sclerosis and retinal diseases including age-related macular degeneration and glaucoma. The ages of AD and control eyes ranged from 62 to 82 years. Control donors’ mean age was 71.75 (N = 4). AD donors’ mean age was 71.20 (N = 5). There was no significant difference between the mean ages of the two groups (Mann–Whitney, *p* = 0.385).

**Table 1:** Demographics of the samples studied

Donor ID	Age	Sex	Primary path Dx	Additional path Dx	A-beta (Thal) (1–5)	Braak stage (1–6)	Neuritic plaque (CERAD, Biel)	Diffuse plaque (CERAD, Biel)
Case1	62	M	AD	–	5	6	Frequent	Frequent
Case2	70	M	AD, DLB	CVD	5	6	Frequent	Frequent
Case3	72	M	AD, LBD	–	5	5	Moderate	Frequent
Case4	70	F	AD	CHR CB Degen	5	6	Frequent	Frequent
Case5	82	M	AD, LBD	CVD, SDH	5	5	Frequent	Frequent
Control1	71	M	N/A	N/A	N/A	N/A	N/A	N/A
Control2	72	F	N/A	N/A	N/A	N/A	N/A	N/A
Control3	73	M	N/A	N/A	N/A	N/A	N/A	N/A
Control4	71	M	N/A	N/A	N/A	N/A	N/A	N/A

### Immunofluorescence dual labelling for IBA-1 and CD68

**Fig. 1:**
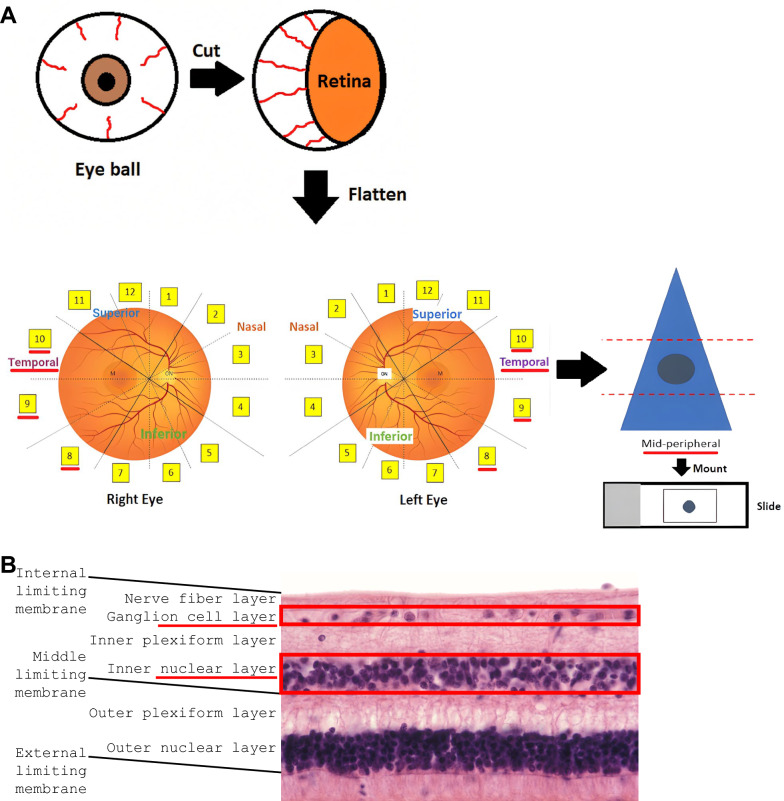
Post-mortem human retinal wholemount preparation and layers in cross sectional view. **A** Schematic displaying the preparation of temporal mid-peripheral wholemount retina punches (3.5 mm in diameter) from human eyes. The figure depicts a punch biopsy in the mid-peripheral of region of the temporal sections 8, 9, and 10. **B** Retinal layers in cross section [24]. The red underlines are indicative of the regions and layers prioritised in the study. Schematics are not to scale

Temporal mid-peripheral wholemount punches (3.5 mm diameter) were processed as described in our earlier studies [22, 23]. Briefly, the samples were prepared for IBA-1 and CD68 immunofluorescence. The CD68 antibody (Cat# ab955, Abcam) was diluted at 1:500 dilution ratio with 3% normal goat serum in 1% Triton X-100 (TX-100) phosphate buffered saline (PBS) (pH 7.2). Free-floating retina punches were incubated at room temperature (RT) for 3 h, followed by overnight incubation at 4 °C. Next, after another series of rinses, punches were incubated with 300 μL of secondary goat anti-mouse Alexa Fluor™ 546 IgG1 antibody (Cat# A21123, Invitrogen) diluted at a 1:400 dilution ratio with 1X PBS (pH 7.2) for 45 min at RT. The punches were then rinsed and incubated with 300 μL of primary rabbit IgG IBA-1 antibody (Cat# 019–19741, Wako) for 2 h at RT. Following another round of rinses, punches were incubated with 300 μL of secondary goat anti-rabbit Alexa Fluor 488 IgG H+L antibody (Cat# A11070, Invitrogen) diluted at a 1:500 dilution ratio with 1X PBS (pH 7.2) for 45 min at RT. Sections were then labelled with DAPI, rinsed in PBS, and coverslipped. Figure 1 summarises the preparation of wholemount retina punches.

### Confocal microscopy and imaging

Wholemount punches were imaged using a Zeiss 800 confocal microscope with Zen Blue 3.6 software. Z-stacks were produced by capturing the NFL to the upper limit of the ONL (Fig. 1B), with digital slices of 1 μm in thickness. The averaging was set to 4X, the mode set to repeat per line, the method set to mean intensity, and the bits per pixels set to 16. The resolution for each stack was set at 2048 × 2048 pixels, with the scaled image size being set at 319.45 μm × 319.45 μm. Nuclear labelling by DAPI was imaged at 405 nm. CD68 labelling by Alexa Fluor™ 546 was imaged at an excitation wavelength of 561 nm, and IBA-1 labelling by Alexa Fluor 488 was imaged at an excitation wavelength of 488 nm.

### Microglia segmentation and analysis with machine learning

Retinal microglia immunolabelled for IBA1 were first segmented using a 3D random forest voxel classifier [25] in Ilastik [19], an interactive machine-learning image analysis software. The random forest classifier labelled each voxel in an image stack by estimating the probabilities that the voxel belonged in user-defined classes. In this case, the classes were defined as ‘microglia,’ ‘vasculature,’ and ‘background.’ The features used by the classifier were voxel intensity, edge, and texture descriptors. Features were extracted using a variety of image filters, such as Gaussian smoothing, Laplacian of Gaussian, Gaussian gradient magnitude, difference of Gaussians, structure tensor eigenvalues, and Hessian of Gaussian eigenvalues, each applied at sigma values of 0.3, 0.7, 1.0, 1.6, 3.5, 5.0, and 10.

**Fig. 2:**
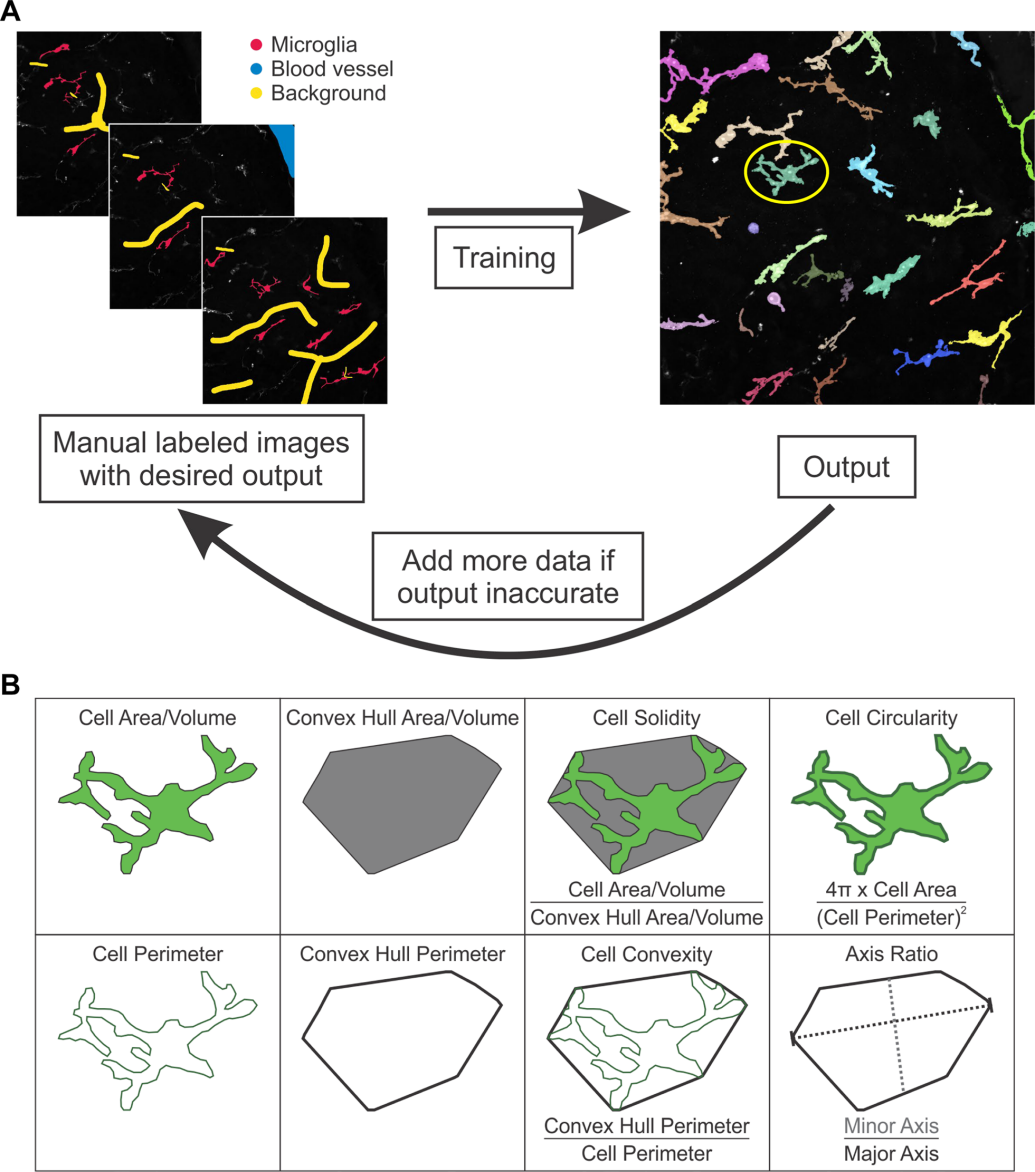
Schematic showing the machine learning pipeline and the different microglia measurements extracted from the analysis. **A** Schematic visualising the microglia labelling using machine learning. In panel A (left), the red label indicates microglia, the blue label indicates blood vessels, and the yellow label indicates the background of the images. The red label was used as inclusion criteria, and the other two labels were used as exclusion criteria for microglia labelling.. The microglia circled in yellow in panel A (right) was used as the microglia model for panel B. **B** Schematic depicting a visual representation of the measurements extracted from our machine learning analysis and manual analysis. Machine learning metrics included 3D metrics like cell volume, convex hull volume, and cell solidity (cell volume divided by convex hull volume), and 2D metrics like cell perimeter, convex hull perimeter, cell convexity, cell circularity, minor axis length, major axis length, and axis ratio. Manual analysis only allowed for 2D metrics to be analysed such as cell area, perimeter, convex hull area, convex hull perimeter, cell solidity (cell area divided by convex hull area) cell convexity, cell circularity, minor axis length, major axis length, and axis ratio. The schematic in B was designed in CorelDRAW X6

For training the classifier, reference labels were generated by expert annotation on image stacks, creating sparse labels for the background, vasculature, and microglia (Fig. 2A). Sparse labels mark only a small portion of voxels in an image, rather than labelling every voxel in the entire image stack. Annotation was performed in 3D and across 22 image stacks. Annotators were blind to the experimental conditions while labelling to ensure unbiased results. A randomly selected subset of microglia from each stack was labelled, providing a representative sample that was satisfactory to train the classifier. Using Ilastik’s live prediction feedback, expert labellers iteratively optimized the segmentation for accuracy across the three classes until the result was satisfactory from visual inspection. Including the third class for vasculature aided the model in converging to an accurate prediction for microglia pixels and helped generate the final microglia labels.

Once trained, the classifier was incorporated into a Python-based pipeline using the scikit-image package [26] to identify individual microglia. This allowed for rapid generation of regions of interest (ROIs) for further morphological analysis. Specifically, the trained random forest classifier provided per-voxel probability values for each label class The microglia probability map was then smoothed with a Gaussian filter (sigma = 1) to reduce noise, and thresholded to isolate high-confidence voxels representing microglia. The threshold value was determined using the scikit-image implementation of the Otsu method. To improve segmentation accuracy, voxels with a high probability of belonging to the vasculature class were excluded as vascular structures fluoresced in the same channel used to image the IBA1 stain, potentially confounding the microglia labelling. This step removed most false positives for the final microglia segmentation. The remaining probability map was binarized, and the Sobel edge detection method was applied. After this step, the binary image and edges were used in a watershed algorithm (scikit-image) to separate individual microglia. ROIs were refined by applying a size filter that removed labels smaller than typical microglia to minimize any remaining false positives. After the automated processing, each microglia ROI was inspected by expert annotators to identify and exclude any remaining false positives or low-quality segmentations from the pipeline. No microglia met these exclusion criteria.

Morphological measures included cell count density, cell volume, cell perimeter, convex hull volume, convex hull perimeter, cell solidity, cell convexity, cell circularity, minor axis length, major axis length, and axis ratio. For metrics requiring two dimensions (e.g., cell perimeter, convex hull perimeter, cell convexity, cell circularity, minor axis length, major axis length, and axis ratio), a z-projection of the microglia ROI was used. Detailed descriptions of these parameters are provided in the study by Leyh et al. [27], with a summary of calculations in Fig. 2B. Student’s t-tests were used to analyze statistical differences between control and AD retinal microglia. Normality was tested in each dataset using the Shapiro–Wilk test to determine whether a parametric or non-parametric test was appropriate.

### Manual microglia segmentation and analysis

Another set of confocal Z-stacks images—from the same donor eyes, but from a different field of view of the specimen—was used for the manual analysis. Image J was used for the segmentation. To expedite the time-consuming task of manually labelling microglia stained with IBA-1, Z-projections were used to segment microglia, and 3–4 microglia were randomly selected from each Z-projection. For random selection, a 10 × 10 grid was placed over the Z-projections, labelled 1–100, and a random number generator was used to select a grid. The microglia that coincided with that grid was selected for segmentation, and this random selection was done 3–4 times for each Z-projection. Morphological analysis consisted of 2D metrics such as cell area, perimeter, convex hull area, convex hull perimeter, cell solidity, cell convexity, cell circularity, minor axis length, major axis length, and axis ratio (Fig. 2B). Finally, phagocytic cups—sites of phagocytosis in microglia [28]—were also manually labelled for quantification. T-tests were used to analyse the statistical relationship between control and AD retinal microglia. In each dataset, normality was tested to determine whether to use parametric or non-parametric statistical tests.

### Statistical analysis

All statistical analyses were conducted on GraphPad Prism 10.2.3 Statistics software, with significance being defined as **** = *p* ≤ 0.0001, *** = *p* ≤ 0.001, ** = *p* ≤ 0.01, and * = *p* ≤ 0.05. Datasets with one independent variable utilised either a Student’s t-test or a Mann–Whitney U test. Datasets with two independent variables utilised a two-way ANOVA with Bonferroni’s multiple comparison test. Refer to each experiment’s figure captions to see what type of statistical test was performed. All the graphs displaying data were produced using GraphPad Prism 10.2.3.

## Results

Microglia were characterised from wholemount retina punches from AD and age-matched control donor retinas (Fig. 1). Microglia were labelled with IBA-1, and Z-stacks were taken with confocal microscopy. A set of microglia Z-stacks was then analysed using machine learning (Fig. 2A) and another set of Z-stacks was analysed manually. Calculated metrics are shown in Fig. 2B. Analysing different sets of Z-stacks with different methods (machine learning vs manual) would help strengthen our conclusions regarding retinal microglia morphology and activity state in AD.

### AD retina exhibit lower microglia counts than control retina

**Fig. 3:**
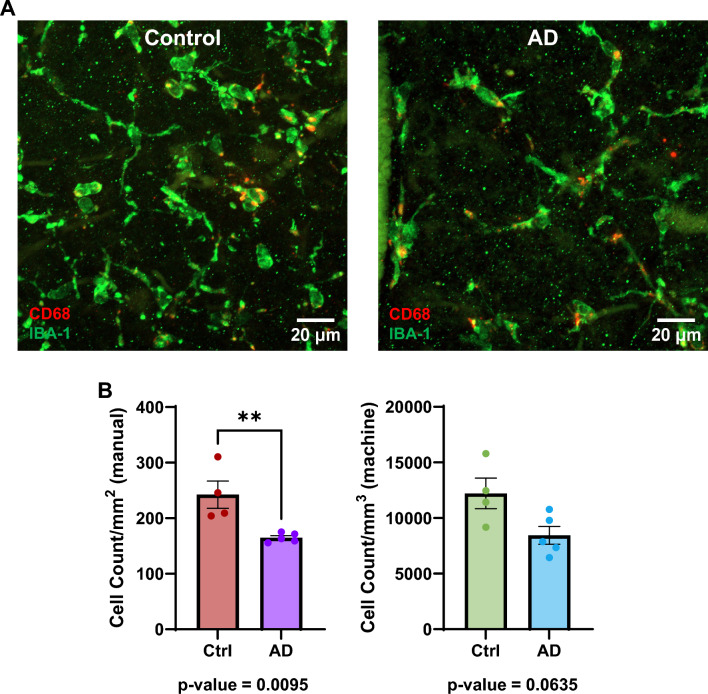
Retinal microglia counts between AD and controls. **A** Example image of an age-matched control (left) and an AD (right) retina with microglia labelled with IBA-1 (green) and CD68 (red) a transmembrane glycoprotein embedded in lysosomal membrane. **B** Column graphs comparing the average microglia count between the control and AD retina using manual (left) and machine learning (right). Points represent individual subject averages and error bars represent the standard error of the mean. P-values for Mann–Whitney U test are below their respective column graphs. * means *p* ≤ 0.05; **** means *p* ≤ 0.0001. (For manual data: Ctrl, n = 4 subjects; AD, n = 5 subjects) (For machine learning data: Ctrl, n = 4 subjects; AD, n = 5 subjects) (Ctrl = Control; AD = Alzheimer’s disease)

To determine if impaired Aβ clearance in the AD retina is due to a reduced population of microglial cells, we imaged IBA-1-labelled microglia (Fig. 3A) and quantified them manually (Fig. 3B left) and through automatic segmentation using machine learning (Fig. 3B right). For all figures, graphs with purple or red columns were from manually segmented data, and graphs with blue or green columns were from automatically segmented data using machine learning. The manually segmented method showed lower microglia counts in the AD retina (manual control: 242.4 ± 24.50 count/mm^2^ (mean ± SEM); manual AD: 165.0 ± 3.633 count/mm^2^ (mean ± SEM); *p* = 0.0095), whereas the machine learning segmented method showed a trend of decreased microglia count per mm^3^ (machine control: 12,213 ± 1378 count/mm^3^ (mean ± SEM); machine AD: 8441 ± 799.6 count/mm^3^ (mean ± SEM); *p* = 0.0635).

### AD retinas exhibit larger microglia than control counterparts

**Fig. 4:**
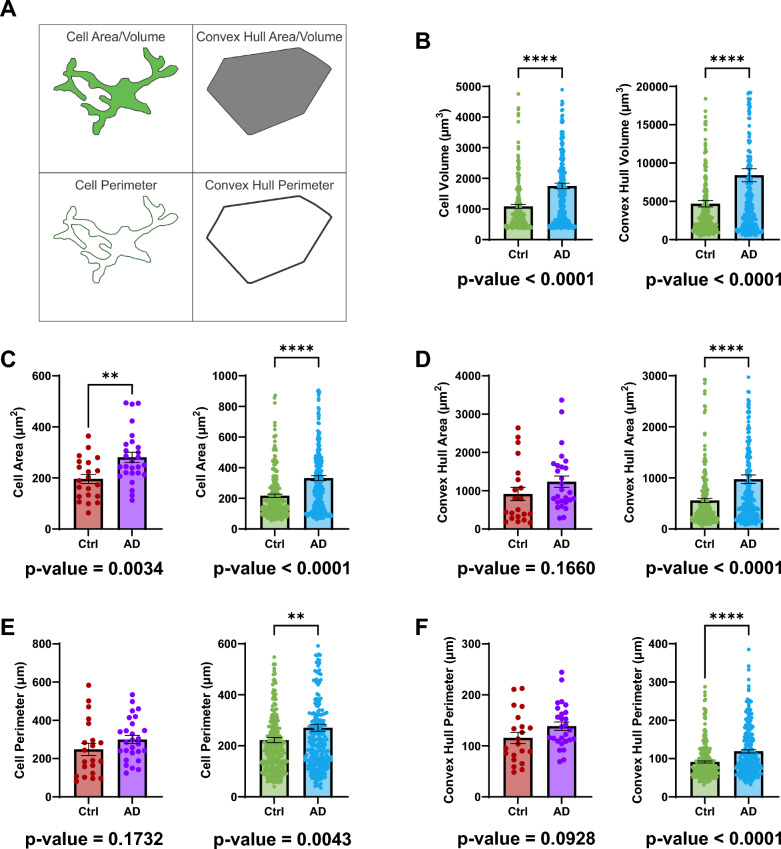
Retinal microglia size between AD and controls. **A** A schematic depicting the parameters in the column graphs B–F. **B** Column graphs comparing the average microglia volume (left) and convex hull volume (right) between control and AD retina using machine learning. **C** Column graphs comparing the average microglia area between control and AD retina using manual analysis (left) or machine learning (right). **D** Column graphs comparing the average microglia convex hull area between control and AD retina using manual analysis (left) or machine learning (right). **E** Column graphs comparing the average microglia perimeter between control and AD retina using manual analysis (left) or machine learning (right). **F** Column graphs comparing the average microglia convex hull perimeter between control and AD retina using manual analysis (left) or machine learning (right). Points represent individual microglia and error bars represent standard error of the mean. P-values for Mann–Whitney U test are below their respective column graphs. * means *p* ≤ 0.05; ** means *p* ≤ 0.01; **** means *p* ≤ 0.0001. (For manual data: Ctrl, n = 21 cells (4 subjects); AD, n = 27 cells (5 subjects)) (For machine learning data: Ctrl, n = 278 cells (4 subjects); AD, n = 268 cells (5 subjects)) (Ctrl = Control; AD = Alzheimer’s disease)

To investigate whether impaired activation of microglial cells contributes to Aβ clearance in the AD retina, we analysed their morphology, which is indicative of microglia activation states. Specifically, we examined microglia size by measuring cell area or volume manually and via machine learning, as well as cell perimeter and their convex hull equivalents. We first studied the size of microglia by measuring the cell area or volume analysed manually and via machine learning respectively, and the cell perimeter and their convex hull equivalents (Fig. 4A). Cell volume (Fig. 4B, left); machine control: 1085 ± 64.56 μm^3^ (mean ± SEM); machine AD: 1754 ± 90.67 μm^3^ (mean ± SEM); *p* < 0.0001) and area (Fig. 4C, left); manual control: 196.1 ± 17.70 μm^2^ (mean ± SEM); manual AD: 280.7 ± 19.81 μm^2^ (mean ± SEM); *p* = 0.0034) (Fig. 4C, right); machine control: 2017.1 ± 11.16 μm^2^ (mean ± SEM); machine AD: 332.1 ± 16.69 μm^2^ (mean ± SEM); *p* < 0.0001) were all larger in AD retinal microglia. Though the manual perimeter was not significantly different between control and AD, there was a trend of larger perimeters in AD (Fig. 4E, left); manual control: 248.2 ± 32.11 μm (mean ± SEM); manual AD: 299.9 ± 21.54 μm (mean ± SEM); *p* = 0.1732), the machine learning analysed perimeter showed larger microglia in the AD retina (Fig. 4E, right); machine control: 222.5 ± 10.12 μm (mean ± SEM); manual AD: 270.6 ± 13.40 μm (mean ± SEM); *p* = 0.0043). The convex hull measurements largely follow the cell area/volume measurements and will be primarily used to calculate the morphological features in the following sections.

### No morphological differences between retinal microglia of AD and controls

**Fig. 5:**
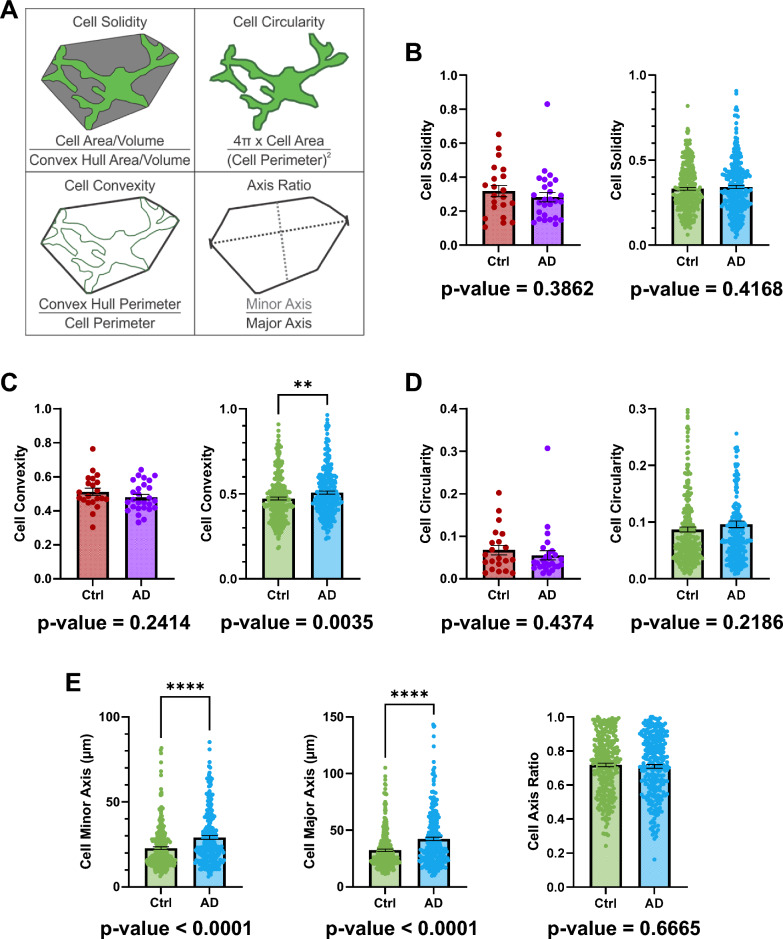
Retinal microglia morphology between AD and controls. **A** A schematic depicting the parameters in the column graphs B–E. **B** Column graphs comparing the average microglia solidity between control and AD retina using manual analysis (left) or machine learning (right). **C** Column graphs comparing the average microglia convexity between control and AD retina using manual analysis (left) or machine learning (right). **D** Column graphs comparing the average microglia circularity between control and AD retina using manual analysis (left) or machine learning (right). **E** Column graphs comparing the average microglia minor axis length (left), major axis length (middle), and axis ratio (right) between control and AD retina using machine learning. Points represent individual microglia and error bars represent standard error of the mean. *P*-values for Mann–Whitney U test are below their respective column graphs. ** means *p* ≤ 0.01; **** means *p* ≤ 0.0001. (For manual data: Ctrl, n = 21 cells (4 subjects); AD, n = 27 cells (5 subjects)) (For machine learning data: Ctrl, n = 278 cells (4 subjects); AD, n = 268 cells (5 subjects)) (Ctrl = Control; AD = Alzheimer’s disease)

To further investigate the morphology of microglia, we analysed their ramification by calculating the cell solidity, circularity, convexity, and axis ratio as shown in Fig. 5A. These calculated values range from 0 to 1. Cell solidity and convexity are related to the microglia’s degree of ramification or amoeboid-ness; a value closer to 1 suggests closer to an amoeboid shape and reduced ramification. Cell circularity and axis ratio are related to the microglia’s spatial symmetry or directionality; a value closer to 1 suggests closer to a circle. We found that there was no difference between AD and control retinal microglia in cell solidity (Fig. 5B, left); manual control: 0.3189 ± 0.03319 (mean ± SEM); manual AD: 0.2820 ± 0.02784 (mean ± SEM); *p* = 0.3862) (Fig. 5B, right); machine control: 0.3321 ± 0.008101 (mean ± SEM); manual AD: 0.3424 ± 0.009785 (mean ± SEM); *p* = 0.4168), convexity (Fig. 5C, left); manual control: 0.5122 ± 0.02150 (mean ± SEM); manual AD: 0.4807 ± 0.01631 (mean ± SEM); *p* = 0.2414) (Fig. 5C, right); machine control: 0.4731 ± 0.008217 (mean ± SEM); machine AD: 0.5075 ± 0.008643 (mean ± SEM); *p* = 0.0035), and circularity (Fig. 5D, left); manual control: 0.06778 ± 0.01121 (mean ± SEM); manual AD: 0.05534 ± 0.01094 (mean ± SEM); *p* = 0.4374) (Fig. 5C, right); machine control: 0.08705 ± 0.004478 (mean ± SEM); machine AD: 0.09625 ± 0.005982 (mean ± SEM); *p* = 0.2186).

We also assessed whether the microglia were more elongated in one group by calculating the axis ratio (Fig. 5A, bottom left panel). There was no difference in the axis ratio between retinal AD and control retinal microglia (Fig. 5E, right); machine control: 0.7182 ± 0.01047 (mean ± SEM); machine AD: 0.7097 ± 0.01101 (mean ± SEM); *p* = 0.6665).

### Larger microglia seen in AD retina are restricted to CD68 + cells

**Fig. 6:**
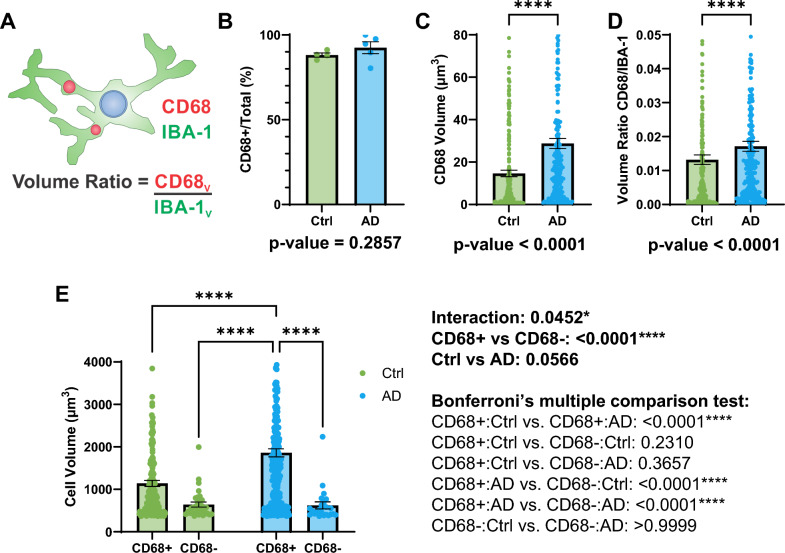
Colocalization of microglia along with lysosome marker CD68 between AD and control retinas. **A** Schematic of a microglia containing lysosomes (CD68) and depicting a visual representation for the values in the column graphs B–y. **B** Column graph comparing the proportion of microglia that are CD68+ between control and AD retina using machine learning. **C** Column graph comparing the average CD68 volume per microglia between control and AD retina using machine learning. **D** Column graph comparing the ratio of CD68 to IBA-1 signal per microglia between control and AD retina using machine learning. **E** Column graph comparing the average cell volume in CD68+ and CD68- microglia between control and AD retina using machine learning. Column graphs and error bars represent the mean and standard error of the mean respectively. Individual data points represent image averages for B or individual cells for C–D. *P*-values for Mann–Whitney U test or two-way ANOVA are below (B–D) or to the right (E) of their respective column graphs. **** means *p* ≤ 0.0001; Mann–Whitney U test for B–D and two-way ANOVA with Bonferroni’s multiple comparison test for E. (For B: Ctrl, n = 4 subjects; AD, n = 5 subjects) (For C–D: Ctrl, n = 278 cells (4 subjects); AD, n = 268 cells (5 subjects)) (For E: CD68+ : Ctrl, n = 247 cells (4 subjects); CD68-: Ctrl, n = 31 cells (4 subjects); CD68+ : AD, n = 245 cells (5 subjects); CD68-: AD, n = 23 cells (5 subjects)) (Ctrl = Control; AD = Alzheimer’s disease; CD68V = CD68 volume; IBA-1 V = IBA-1 volume)

To investigate the potential reasons for the observed increase in microglia size, we hypothesized that larger microglia might contain more lysosomes due to the increased demand for clearing Aβ. To test this, we labeled CD68, a transmembrane glycoprotein present in lysosomal membranes, with Alexa Fluor 546. Figure 6A depicts a schematic of the lysosomes in the microglia. First, we observed that the proportion of the microglia containing CD68 labelling (CD68+) was simlar between the AD and control retina (Fig. 6B; machine control: 88.12 ± 1.229 (mean ± SEM); machine AD: 92.46 ± 3.488% (mean ± SEM); *p* = 0.2857). Interestingly, a higher volume of CD68 labelling was found on average in each retinal microglia in AD compared to control (Fig. 6C; machine control: 14.66 ± 1.533 μm^3^ (mean ± SEM); machine AD: 28.76 ± 2.365 μm^3^ (mean ± SEM); *p* < 0.0001), and this increase persisted even after normalising to the microglia volume (Fig. 6D; machine control: 0.01320 ± 0.001396 (mean ± SEM); machine AD: 0.01715 ± 0.001451 (mean ± SEM); *p* < 0.0001). Furthermore, the larger microglia seen in the AD retina were restricted to the CD68 + population, and were larger than CD68- microglia (in AD) as well as the CD68 + microglia (in controls) (Fig. 6E; CD68 + control: 1141.293 ± 71.504 μm^3^ (mean ± SEM); CD68- control: 640.486 ± 60.079 μm^3^ (mean ± SEM); CD68 + AD: 1860.064 ± 96.137 μm^3^ (mean ± SEM); CD68- AD: 622.622 ± 83.165 μm^3^ (mean ± SEM); *p*-values found in Fig. 6E (right)).

### Microglia phagocytic cups were similar between AD and control retina

**Fig. 7:**
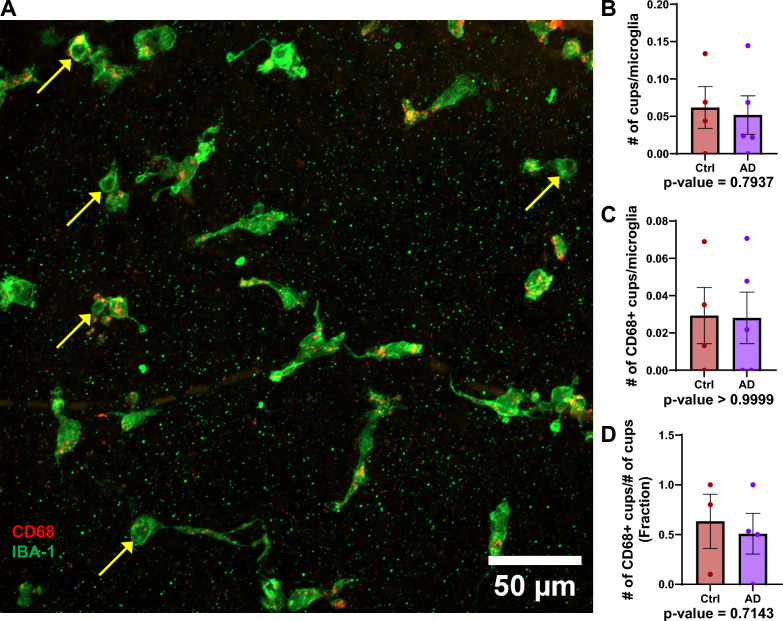
Differences in retinal microglia with respect to phagocytic cups between AD and controls. **A** Example image of an AD retina with microglia labelled with IBA-1 (green) and CD68 (red) a transmembrane glycoprotein embedded in lysosomal membrane, with phagocytic cups indicated with yellow arrows. **B** Column graph comparing the average number of cups/microglia between control and AD retina using manual analysis. **C** Column graph comparing the average number of CD68 + cups/microglia between control and AD retina using manual analysis. **D** Column graph comparing the fraction of CD68 + cups/total number of cups between control and AD retina using manual analysis. Points represent individual image averages and error bars represent standard error of the mean. *P*-values for Mann–Whitney U test are below their respective column graphs. (For B–D: Ctrl, n = 4 subjects; AD, n = 5 subjects) (Ctrl = Control; AD = Alzheimer’s disease)

We also quantified phagocytic cups, which are bowl-like structures formed by microglia during the process of phagocytosis. Figure 7A is a representative image showing phagocytic cups indicated with yellow arrows. The number of phagocytic cups may suggest the level of activation of microglia as well. We did not identify any difference in the average number of cups per microglia (Fig. 7B; manual control: 0.06170 ± 0.02799 cups/microglia (mean ± SEM); manual AD: 0.05172 ± 0.02574 cups/microglia (mean ± SEM); *p* = 0.7937), the average number of CD68 + cups per microglia (Fig. 7C; manual control: 0.02930 ± 0.01507 CD68 + cups/microglia (mean ± SEM); manual AD: 0.03092 ± 0.01383 CD68 + cups/microglia (mean ± SEM); p > 0.9999), and the proportion of phagocytic cups containing CD68 (Fig. 7D; manual control: 0.6333 ± 0.2728 (mean ± SEM); manual AD: 0.5083 ± 0.2043 (mean ± SEM); *p* = 0.7143).

## Discussion

The role of microglia in AD pathophysiology is complex due to their diverse phenotypes and various activation pathways [29]. In the early stages of disease progression, microglia are considered beneficial as they proliferate, change morphology, and phagocytose harmful substances, including Aβ plaques and dead cells, after the surveillance and perception of neuronal damage in their microenvironment [16, 23]. Furthermore, microglia have been shown to provide protection and facilitate the repair of damaged neurons by secreting trophic factors [16]. However, activated microglia also trigger inflammatory pathways, resulting in the production of cytokines. This promotes further Aβ production, leading to excessive accumulation of harmful substances, reduces the release of trophic factors, and accelerates neuronal apoptosis and degenerative changes during the later stages of disease progression [30, 31]. Moreover, it has been shown that pTau could also negatively affect the phagocytic function of microglia by promoting their senescence, which leads to their dysfunctional phagocytic activity, exacerbates the accumulation of tau proteins and Aβ, and promotes neuronal damage and disease progression [32–34].

In this study, we explored the differences in the microglial populations between AD and healthy controls in the inferior and superior temporal regions of the inner retina. Our study showed a significantly reduced microglia population in AD retina compared to controls in the manual data (*p* = 0.0095). There was similar difference in the machine learning data but without reaching statistical significance (*p* = 0.0635). This difference may be due to the combination of the use of different fields of view (and thus different images) in the same set of specimens for the manual and machine learning data and the small sample size (n = 4 for control, and n = 5 for AD). Variability was also much lower in the manually obtained AD cell count. Due to this discrepancy between our manual and machine learning data, we cannot rule out the potential for a type I or type II error with our microglia count data. If microglia are indeed decreased in the AD retina, this finding might result from increased senescence and apoptosis of microglia secondary to the presence of tau proteins and neuroinflammation, and the reduction of microglia population could contribute to the decreased clearance of Aβ and NFTs in the neurons.

According to the literature, several studies have reported microgliosis, or an increase in the microglia population, in the AD retina, which contrasts with our findings [11, 35–37]. There may be several factors contributing to this discrepancy. First, most previous studies on the microglia population utilised cross-sections of the donor retina, which may yield different results compared to our study, which used punches of the full thickness retina tissue. Secondly, Koronyo et al. [37] suggested that their mapping of IBA-1 distribution was significantly greater in the C subregion, while most of our data was collected from the mid-peripheral region of the retina. Their normalised data, adjusted for retinal thickness, showed the most significant microgliosis in the mid-peripheral region in the superior temporal, whereas the majority of our samples were collected from the mid-peripheral region in the inferior temporal region. Thirdly, it should be noted that the donor demographic differs significantly between studies. In their study, 55% and 67% of the donors in the mild cognitive impairment (MCI) and AD groups, respectively, were Braak stage V–VI, while 100% of the AD donors in our study were Braak stage V–VI. One possible explanation is that the microglia population may change with the disease progression, potentially being higher in the earlier stage and decreasing as disease severity increases. In the initial stages of the disease, microglia may be recruited to clear Aβ, resulting in an increase in number and activation. However, as the Aβ load becomes too high for the resident microglia to effectively clear, it may lead to a loss in microglia count and function. In fact, Koronyo et al. [37] demonstrated that a lower proportion of microglia in the AD retina were engaging in Aβ uptake compared to normal cognition controls, implying impaired microglia function.

Although there is limited knowledge about changes in microglia population in AD, research findings from another neurodegenerative disease, dementia with Lewy Bodies (DLB), may provide some insights. DLB has shown increased microglial activation in the early stages of the disease, with significantly reduced transporter protein density indicative of microglia dystrophy in the late stage of the disease [38, 39].

Lastly it is important to consider that most other studies, including Koronyo et al. [37], investigated the microgliosis using the intensity of the immunoreactivity of the IBA-1 marker. This approach differs from counting microglia, as the fluorescent intensity of the marker is not only correlated with the microglia population but also affected by the size of the microglia. In our previous study [11] using retinal cross-sections, we indeed observed that there was increased IBA-1 immunoreactivity in the AD retina. However, this does not directly count the number of individual microglia. The current results show that AD microglia have larger area or volume than the control microglia, which would contribute to observation of increased amount of immunoreactivity.

Our findings revealed that the size of CD68 + /IBA-1 microglia was larger in the AD compared to the controls, whereas the size of the CD68-/IBA-1 microglia was not significantly different between AD and controls. Of note, majority of microglia population were CD68 + in both groups (88% in control and 92% in AD, no significant difference). CD68, a transmembrane glycoprotein embedded in lysosomal membrane, was used as a marker of phagocytic activity. We observed a higher volume of CD68 per microglia in AD, and it was the CD68 + microglia that were enlarged in AD, whereas the CD68- microglia were significantly smaller and similar between AD and control retina. The average measurements were greater in AD microglia than controls for all size parameters (cell area, cell volume, convex hull area, convex hull volume, cell perimeter, convex hull perimeter) in both manual and machine-learning data. The difference was statistically significant in all machine-learning parameters, but only in cell area for the manual parameters. This discrepancy in statistical significance is likely in part due to the difference in the number of measured microglia and the resulting statistical power between the two approaches: the manual measurements are from 3–4 sample microglia in 2D projection images whereas the machine learning measurements are from all microglia in entire z-stacks. There may have also been some sampling bias in the 2D method. The microglia were selected randomly using the grid method; however, the 2D projection step or the grid resolution could have resulted in a size-based bias. In Fig. 4, we note that although the mean values are similar between the manual and automated measurements, the distributions look quite different between the two methods. The machine learning values from all microglia are often long-tail and bottom-heavy distributed, but the manual measurement values from select microglia are more centrally distributed. The range of the values also differ; for example, for Cell Area the machine learning method reports microglia of above 600 µm^2^ and close to 900 µm^2^, but the largest microglia area reported by the manual method is less than 600 µm^2^. On the whole, the raw data points and their mean values in both manual and machine learning measurements demonstrate a consistent trend of larger microglia in AD than in control.

Increase in microglia size may reflect the accumulation of harmful pTau proteins and Aβ in the lysosomes, given the elevated levels of toxic Aβ and pTau present in the AD retina [40, 41], and the increased uptake of Aβ [37] and pTau [42, 43] by microglia in these conditions. Furthermore, it is established that the soma of microglia become enlarged when they are activated in an amoeboid morphology, as opposed to the ramified shape with a smaller soma. The proportion of CD68 + microglia is similar in both AD and control retinas at approximately 90%, and the number of CD68 + and CD68- phagocytic cups per microglia also does not differ between the two groups. However, increased CD68 immunoreactivity in individual microglia within the AD group suggests a higher level of activation in response to AD pathology. This highlights subtle but potentially important differences in microglial behavior between AD and control retinas. Our study provides a comprehensive, multi-faceted characterization of microglial activation. Another study showed that microgliosis occurs during AD, with increased Aβ deposits causing an increase in the number of microglia, while also a much fewer number of them are involved in Aβ uptake compared with normal controls [37]. Although many studies have examined the evidence of upregulated microglial responses in retina tissues from AD patients, our understanding of retinal microglial responses remains limited. Further research is needed to investigate the mechanisms and timeline of the changes in microglial population and size.

Microglia exhibit a variety of different morphologies that are associated with distinctive functions [44]. Their morphology has been shown to drastically change in different parts of the brain and with ageing [45]. Neurodegenerative diseases such as AD have also been associated with different microglia morphology [45]. The most abundant type of microglia morphology in a healthy adult CNS is the ramified phenotype, typically recognised with a small microglial soma connected to several long and ramified processes that are usually several times larger than the cell body itself [46]. The ramified microglia are responsible for surveillance during steady-state conditions [47], and they utilise their highly dynamic and mobile branches to survey their surroundings and detect any changes in the microenvironment. Once neuronal damage or toxic substances have been sensed, ramified microglia undergo an activation process and become ameboid-shaped with round and large cell bodies devoid of cell processes [44]. Unlike the ramified microglia for surveillance, the amoeboid morphology may allow for better mobility to the injured brain area and capacity for phagocytosis [47]. It has been shown that the morphological change between ramified to amoeboid microglia takes only 30–60 min in the brain [48, 49]. Other distinctive morphologies include hypertrophic/hyper-ramified, rod, dystrophic, and satellite microglia [44].

Previous studies have reported that there was an increased level of amoeboid microglia in the hippocampus and cerebral cortex in the AD brain [38, 50], and other studies have shown increased rod, hypertrophic/hyper-ramified, and dystrophic microglia during AD pathogenesis [44, 45]. However, there is a lack of reports of similar findings in the AD retina, which provides the rationale for this study, which focuses on the morphology of retinal IBA-1 labelled microglia within the AD eye of post-mortem donors using confocal microscopy. We investigated morphological features such as cell solidity, convexity, circularity, and phagocytic cup count between AD and control, and found no significant difference between the groups. It is worthwhile to mention that the microglia morphological changes are associated with specific retinal locations and the progression of neurodegenerative disease [45], and therefore, it is possible that the microglia morphology may be different between the inferior temporal of the mid-peripheral retina we used and other retinal regions. It is also important to consider that our study used AD donor tissues from Braak stage V–VI. Therefore, future studies investigating the microglia morphology in other regions of the retina and at different disease stages are warranted.

## Conclusions

Here, we studied cell solidity, convexity, and circularity, foundational parameters to examine cell morphology, however it should be noted that Choi et al. utilised supervised machine learning with support vector machine to accurately and reproducibly quantify and categorise five distinctive retinal microglial cell morphologies [51]. Their group reported statistically significant group differences among morphotypes with shape descriptors, which enables accurate description of the entire population of microglial cells [51].

Our study highlighted that the increased size of microglia in AD eyes may be a valuable addition to the diagnostic toolkit for ocular in-vivo imaging of AD. Wahl et al. demonstrated the use of sensorless adaptive optics (SAO) for non-invasive single-cell imaging in the retina of wild-type (C57BL/6J) and transgenic mice with green fluorescent protein (GFP)-labelled microglia (Cx3cr1-GFP). This high-resolution technique allowed for clear observation of the dynamics of microglial branches [52]. The ability to count and measure retinal microglia in vivo will enhance our understanding of the changes that occur with AD progression and the efficacy of Aβ-lowering drugs. Coupled with studies revealing the presence of Aβ in the retina and tears of AD patients [53], the role of ocular non-invasive techniques in diagnosing and monitoring AD progression may become crucial in the future.

## Data Availability

Data is provided within the manuscript.

## Abbreviations

Aβ: Amyloid-β
AD: Alzheimer’s disease
CD68: Cluster of differentiation 68
CNS: Central nervous system
CT: Computed tomography
Ctrl: Control
DAMPS: Damage-associated molecular patterns
DLB: Dementia with lewy bodies
GFP: Green fluorescent protein
IBA-1: Ionised calcium-binding adaptor molecule 1
MCI: Mild cognitive impairment
MRI: Magnetic resonance imaging
OCT: Optical coherence tomography
PBS: Phosphate buffered saline
PET: Positron emission tomography
pTau: Tau proteins
RT: Room temperature
SAO: Sensorless adaptive optics
SEM: Standard error of the mean
SLO: Scanning laser ophthalmoscopy
TX-100: Triton X-100
VGH: Vancouver General Hospital

## Code availability

Code used for the automated segmentation and analysis of the retinal microglia are available for download on GitHub at https://github.com/Peter-Hogg/Retinal-Microglia-Segmentation. The Ilastik project files are stored on the Open Science Framework at https://osf.io/euspt/?view_only=755c79f38ad245908d3aa8b5620c4863.
